# Survival of Older Patients Who Receive Cardiac Resynchronization Therapy Devices With or Without Defibrillation Capability

**DOI:** 10.1016/j.jacep.2025.08.010

**Published:** 2025-09-24

**Authors:** Samir Saba, Sandeep Jain, Mark Estes, Madhurmeet Singh, Alaa Shalaby

**Affiliations:** Heart and Vascular Institute at the University of Pittsburgh Medical Center, Pittsburgh, Pennsylvania, USA.

**Keywords:** cardiac resynchronization therapy, defibrillator, heart failure, mortality, pacemaker

Cardiac resynchronization therapy (CRT) is an established treatment for patients with advanced heart failure (HF)^1,2^ that can be delivered through a pacemaker (CRT-P) or a defibrillator (CRT-D). Despite significant differences in size, function, cost, and complications between CRT-P and CRT-D,^3^ current guidelines^1^ do not separate indications between these 2 devices. Randomized data comparing CRT-P versus CRT-D recipients show no difference in the primary endpoint of death or HF hospitalization but suggest a trend toward lower mortality with CRT-D.^2^

A randomized controlled trial (RCT) comparing outcomes after CRT-P versus CRT-D is therefore necessary to guide clinical management, especially in elderly patients who have shorter longevity and experience fewer defibrillator shocks.^4^ We previously conducted a pilot RCT to assess the feasibility of enrolling elderly HF patients in a study of CRT-P versus CRT-D,^5^ and we here present the long-term survival of patients in this RCT and its accompanying observational registry (OR).

As previously described^5^, a pilot RCT (Cardiac resynchronization in the elderly; NCT03031847) was conducted between 2017 and 2021 at 4 institutions in the United States. The protocol was approved by the institutional review board of the University of Pittsburgh and of the participating institutions. Eligible study patients met the following inclusion criteria: 1) age ≥75 years; 2) left ventricular ejection fraction ≤35%; 3) QRS width >120 ms; 4) NYHA functional class II, III, or ambulatory IV; and 5) no prior history of ventricular arrhythmia. Participants who declined randomization were followed up as part of the OR.

Of the original cohort of 106 patients, long-term mortality data could be obtained on 88 (83%) patients. Of the total cohort, 36 (20 CRT-P, 16 CRT-D) patients were enrolled in the RCT, and 52 (17 CRT-P, 34 CRT-D, 1 declined procedure after enrollment) in the OR. Patients were followed up to the endpoint of all-cause mortality or last follow-up visit through May 1, 2025. When possible, the cause of death was classified as cardiac or noncardiac, and further as arrhythmic or nonarrhythmic.

A comparison of baseline characteristics and outcomes was carried between CRT-P and CRT-D recipients as well as between RCT and OR patients, to address any safety concerns linked to enrollment into this RCT. Time to all-cause mortality was calculated using multivariable Cox survival models to compare overall survival between groups, adjusting for unbalanced covariates (all variables with *P* < 0.10 by univariable analyses).

The participants (age 81 ± 5 years, QRS 164 ms ± 27 ms, ejection fraction 25% ± 5%) were mainly white men, and most were insured by Medicare. The burden of comorbidities showed no noticeable differences between participants in the RCT versus the OR, except for a higher prevalence of ischemic cardiomyopathy (*P* = 0.045), Medicare as a primary insurance (*P* < 0.001), and a trend toward lower body mass index (*P* = 0.09) in the OR compared with the RCT cohort.

There were 49 deaths in 88 participants over a median follow-up time of 3.4 years: 42% in the RCT and 65% in the OR groups (*P* = 0.032). Mortality rates in the RCT cohort trended lower than in the OR cohort (unadjusted HR: 0.55; 95% CI: 0.30–1.02; *P* = 0.058). When we adjusted for differences in the type of cardiomyopathy, primary insurance, and body mass index, patients in the RCT group had significantly lower mortality than did those in the OR group (adjusted HR: 0.39; 95% CI: 0.17–0.88; *P* = 0.024 (Figure 1A).

We compared mortality in the overall cohort by the type of device implanted. There were 22 deaths in the CRT-P group and 26 in the CRT-D group (59% vs 52%, *P* = 0.52). Time to death was similar between the CRT-P and CRT-D cohorts (unadjusted HR: 1.27; 95% CI: 0.72–2.24; *P* = 0.41) even after adjustment for unbalanced variables (adjusted HR: 0.96; 95% CI: 0.52–1.75; *P* = 0.89 (Figure 1B). Similar results were noted when we examined the survival of CRT-P versus CRT-D recipients in RCT patients only (HR: 1.35; 95% CI: 0.46–3.97; *P* = 0.59).

The cause of death could be adjudicated in 41 of 49 (84%) patients. The cause of death was cardiac in 16 and noncardiac in 25 patients. Refractory ventricular arrhythmias resulted in the death of 2 patients, both implanted with a CRT-D.

Our pilot yielded important results. First, we found that older patients referred for CRT are willing to enroll in an RCT of CRT-P versus CRT-D and that enrollment did not have a negative impact on their mortality. Equally important is the finding that older patients with no prior history of sustained ventricular arrhythmias had comparable all-cause mortality after implantation with CRT-P or CRT-D, over a median follow-up time of 3.4 years in this pilot trial. These results suggest that the smaller and less costly CRT-P may be favorably considered in this population. These results should be confirmed in a larger multicenter RCT before this practice can be clinically adopted.

## Figures and Tables

**FIGURE 1:**
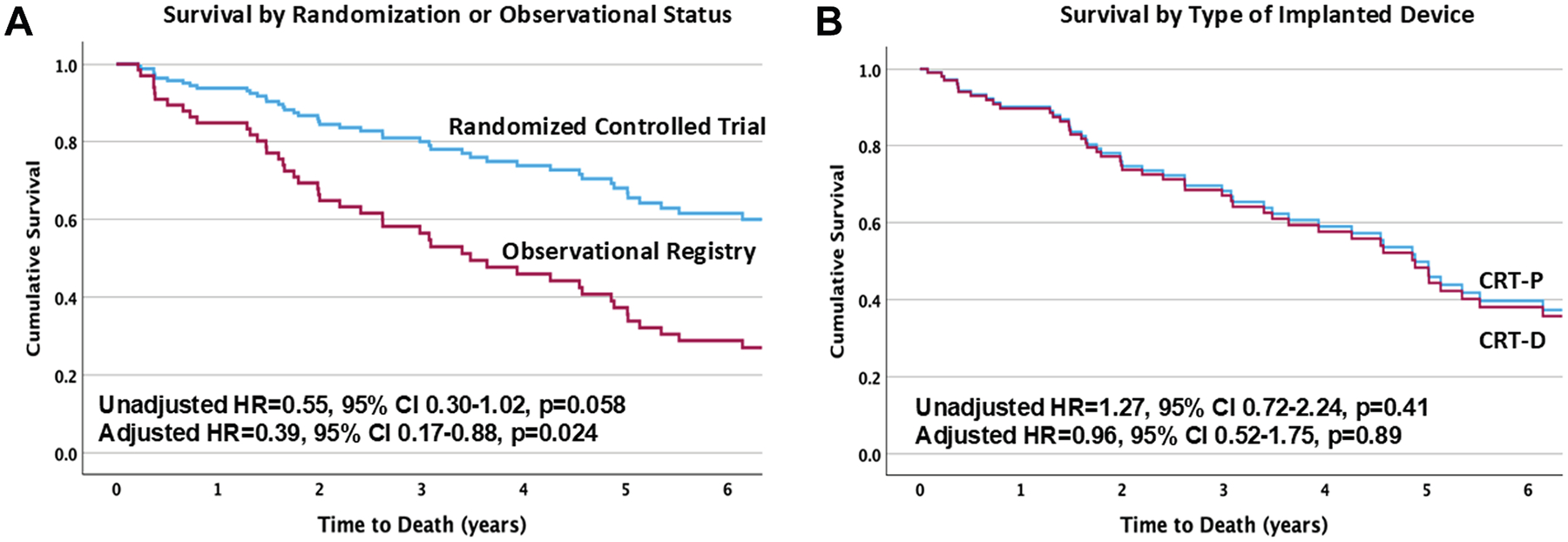
Survival of Older CRT Device Recipients (A) Survival of patients enrolled in the randomized controlled trial versus the observational registry. (B) Survival of CRT device recipients by type of implanted device: cardiac resynchronization therapy—pacemaker (CRT-P) versus cardiac resynchronization therapy—defibrillator (CRT-D).
